# TrajectoryNAS: A Neural Architecture Search for Trajectory Prediction

**DOI:** 10.3390/s24175696

**Published:** 2024-09-01

**Authors:** Ali Asghar Sharifi, Ali Zoljodi, Masoud Daneshtalab

**Affiliations:** 1 School of Innovation, Design and Technology (IDT), Mälardalen University, 72123 Västerås, Swedenali.zoljodi@mdu.se (A.Z.); 2 Department of Computer Systems, Tallinn University of Technology, 19086 Tallinn, Estonia

**Keywords:** autonomous driving, neural architecture search, trajectory prediction, 3D point cloud

## Abstract

Autonomous driving systems are a rapidly evolving technology. Trajectory prediction is a critical component of autonomous driving systems that enables safe navigation by anticipating the movement of surrounding objects. Lidar point-cloud data provide a 3D view of solid objects surrounding the ego-vehicle. Hence, trajectory prediction using Lidar point-cloud data performs better than 2D RGB cameras due to providing the distance between the target object and the ego-vehicle. However, processing point-cloud data is a costly and complicated process, and state-of-the-art 3D trajectory predictions using point-cloud data suffer from slow and erroneous predictions. State-of-the-art trajectory prediction approaches suffer from handcrafted and inefficient architectures, which can lead to low accuracy and suboptimal inference times. Neural architecture search (NAS) is a method proposed to optimize neural network models by using search algorithms to redesign architectures based on their performance and runtime. This paper introduces TrajectoryNAS, a novel neural architecture search (NAS) method designed to develop an efficient and more accurate LiDAR-based trajectory prediction model for predicting the trajectories of objects surrounding the ego vehicle. TrajectoryNAS systematically optimizes the architecture of an end-to-end trajectory prediction algorithm, incorporating all stacked components that are prerequisites for trajectory prediction, including object detection and object tracking, using metaheuristic algorithms. This approach addresses the neural architecture designs in each component of trajectory prediction, considering accuracy loss and the associated overhead latency. Our method introduces a novel multi-objective energy function that integrates accuracy and efficiency metrics, enabling the creation of a model that significantly outperforms existing approaches. Through empirical studies, TrajectoryNAS demonstrates its effectiveness in enhancing the performance of autonomous driving systems, marking a significant advancement in the field. Experimental results reveal that TrajcetoryNAS yields a minimum of 4.8 higger accuracy and 1.1* lower latency over competing methods on the NuScenes dataset.

## 1. Introduction

Predicting future actions or states of objects around an intelligent system, such as an autonomous driving (AD) vehicle, is crucial in preventing disasters or crashes. Driving in the real world is a stochastic process due to the presence of other vehicles and pedestrians that can take their next step, resulting in accidents or congestion. AD systems require the crucial ability to predict the trajectory of surrounding objects [1,2,3]. Predicting accurately the trajectory of surrounding objects is important in simultaneous localization and mapping (SLAM) because it provides crucial information about static and dynamic objects and allows for the refinement of object locations based on these predicted trajectories [4]. To perform the task of predicting in self-driving vehicles, 2D and 3D data can be utilized. 3D data can usually be represented in different formats, including depth images, point clouds, meshes, and volumetric grids. The optical camera is usually good for classification tasks such as distinguishing the type of surrounding objects or detecting lane markers or traffic signs. While the performance of measuring distances and velocities is rather weak, this information can be retrieved well from radars. LIDARs are complementary to the other two sensors, showing competitive results. Distances and velocities can be estimated with very high accuracy. Therefore, it is the preferred representation for many scene-understanding-related applications such as autonomous driving and robotics.

Our paper presents TrajectoryNAS, an application-specific Neural Architecture Search (NAS) that aims to create a trajectory model with high accuracy and minimum displacement errors, both final and average (FDE and ADE (Section 4.2)). Our empirical studies reveal that accurate object detection is crucial to achieving precise trajectory predictions. Therefore, TrajectoryNAS is designed to localize objects with a minimum error and improve the accuracy of final trajectory predictions. Additionally, to minimize the time required for inference, the final objective of TrajectoryNAS is to reduce the model latency.

In conclusion, our contributions to this challenge can be summarized as follows:

- **Trajectory Prediction NAS:** TrajectoryNAS is a novel trajectory prediction for autonomous driving, being the first to implement neural architecture search (NAS) in an end-to-end manner. It integrates object detection, tracking, and predicting, addressing the complex interdependencies among these tasks and the challenges of point-cloud processing.
- **Hybrid Exploration and Exploitation:** We introduce a two-step process to efficiently handle the computational demands of NAS on large datasets. This approach first explores architectures using a mini dataset, which is 10× faster than the complete dataset, and then trains the selected architecture on the full dataset (exploitation), ensuring both scalability and accuracy.
- **Multi-Objective Architecture Search:** We introduce a multi-objective energy function to assess the proposed architecture in both an accuracy and latency manner.

## 2. Related Works

### 2.1. Trajectory Prediction

**Figure 1:**
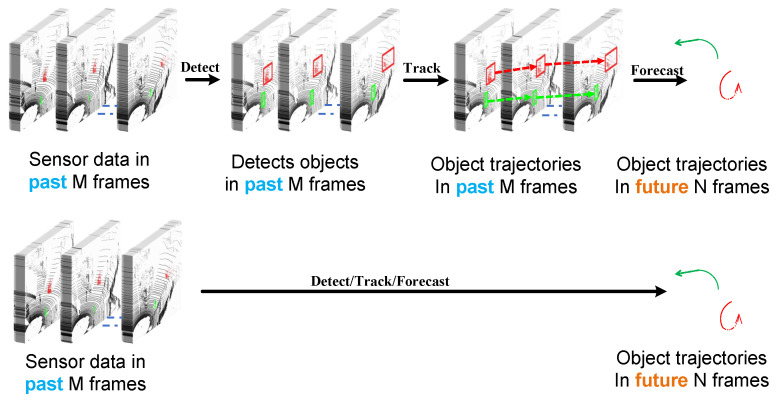
(Top Row) Cascade methods that independently address detection, tracking, and predicting, they inherently carry the risk of compounding errors throughout the pipeline. This originates from—each sub-module’s assumption of receiving perfect input, which rarely holds true in real-world applications. Consequently, errors introduced in earlier stages propagate and magnify downstream, potentially leading to inaccurate final outcomes. (Bottom Row) End-to-end methods that predict future movement directly from raw data, enabling end-to-end training and benefiting from the joint optimization of object detection, tracking, and prediction tasks.

In this section, we provide a brief overview of the literature focused on predicting trajectories using point-cloud data. We begin by exploring cascade approaches (traditional approaches). In these approaches, the output of a detector serves as input to a tracker. The tracker’s output is then used by a trajectory-predicting algorithm to estimate the anticipated movements of traffic participants in the upcoming seconds as in Figure 1 (top row). Following that, the state-of-the-art approaches that do detection, tracking, and predicting in an end-to-end manner are reviewed, depicted in Figure 1 (bottom row).

#### 2.1.1. Cascade Approaches

Traditional self-driving autonomy decomposes the problem into three subtasks (object detection, object tracking, and motion prediction) and relies on independent components that perform these subtasks sequentially. These modules are usually learned independently, and uncertainty is usually propagated [1]. In these methods, it is assumed that the exact paths taken by the agents are known. By examining the trajectory data over a short period of time, predictions can be made for future moments. For instance, the NuScenes [5] and Argoverse [6] datasets provide trajectories and their corresponding labels for this purpose.

Many of the approaches presented in the literature are based on neural networks that use recurrent neural networks (RNNs), which explicitly take into account a history composed of the past states of the agents [7]. In RNNs and their variants, memory is a single hidden state vector that encodes all the temporal information. Thus, memory is addressable as a whole, and it lacks the ability to address individual elements of knowledge [3]. Ref. [3] presents the memory-augmented neural trajectory predictor (MANTRA). In this model, an external, associative memory is trained to store useful and non-redundant trajectories. Instead of a single hidden representation addressable as a whole, the memory is element-wise addressable, permitting selective access to only relevant pieces of information at runtime.

Spatial and temporal learning will be two key components in prediction learning. Ignoring either information will lead to information loss and reduce the model’s capability of context learning. Consequently, researchers are focusing on jointly learning RNN spatial and temporal information. Ref. [8] utilize rasterization to encode both the agents and high-definition map details, transforming corresponding elements such as lanes and crosswalks into lines and polygons of diverse colors. However, the rasterized image is an overly complex representation of environment and agent history and requires significantly more computation and data to train and deploy. In an effort to address this, VectorNet [9] proposes a vector representation to exploit the spatial locality of individual road components with graph neural networks. LaneConv [10] constructs a lane graph from vectorized map data and proposes LaneGCN to capture the topology and long dependency of the agents and map information. Both VectorNet [9] and LaneConv [10] can be viewed as extensions of graph neural networks in prediction with a strong capability to extract spatial locality. Nevertheless, both works fail to fully utilize the temporal information of agents with less focus on temporal feature extraction. In order to combine spatial and temporal learning in a flexible and unified framework, Ref. [11] proposes temporal point-cloud networks (TPCN). TPCN models the prediction learning task as joint learning between a spatial module and a temporal module.

Across a range of visual benchmarks, transformer-based models exhibit comparable or superior performance when compared to other network types like convolutional and recurrent neural networks [12]. This trend extends to trajectory prediction as well. Ref. [13] proposes a new transformer that simultaneously models the time and social dimensions. Their method allows an agent’s state at one time to directly affect another agent’s state in the future. In parallel, Ref. [14] develops an RNN-based approach for context-aware multi-modal behavior forecasting. The model input includes both a road network attention module and a dynamic interaction graph to capture interpretable geometric and social relationships.

As mentioned, cascade approaches in order to trajectory prediction are developed separately from their upstream perception. As a result, their performance degrades significantly when using real-world noisy tracking results as inputs. Ref. [15] presents a novel prediction framework that uses affinity matrices rather than tracklets as inputs, thereby completely removing the chances of errors occurring in data association and passing more information to prediction. To consider this propagation of errors, Ref. [15] applies three types of data augmentation to increase the robustness of prediction with respect to tracking errors. They inject identity switches (IDS), fragments (FRAG), and noise.

#### 2.1.2. End-to-End Approaches

To prevent the propagation of errors and reduce inference time in traditional methods, as they learn independently, researchers [16,17,18,19] attempted to perform detection and tracking in an end-to-end manner. With the same purpose, Ref. [20] proposed a network that parallelized tracking and prediction using a graph neural network (GNN).

To our best knowledge, Fast and Furious (FaF) [21] proposes the first deep neural network capable of jointly performing 3D detection, tracking, and motion prediction using data captured by a 3D sensor. However, Ref. [21] limited its predictions to a mere 1 s duration. In contrast, IntentNet [22] enlarges the prediction horizon and estimates future high-level driver behavior. Ref. [23] moved a step further and performed detection, predicting, and motion planning jointly. Furthermore, Ref. [23] introduces an additional perception loss that encourages the intermediate representations to generate accurate 3D detections and motion prediction. This ensures the interoperability of these intermediate representations and enables significantly accelerated learning. The statistical interconnections among actors are overlooked by all the previously mentioned methods, and instead, they individually predict each trajectory using the provided features. Ref. [2] designed a novel network that explicitly takes into account the interactions among actors. To capture their spatial-temporal dependencies, Ref. [2] proposes a recurrent neural network with a transformer architecture.

Ref. [24] suggests a reversing of the detect-then-forecast pipeline rather than following the conventional sequence of detecting, tracking, and subsequently forecasting objects. Afterward, object detection and tracking are performed on the projected point-cloud sequences to obtain future poses. A notable advantage of this methodology lies in the comprehensive representation of predictions, incorporating details about RNNs and the background and foreground objects existing within the scene. Similarly, in a comparable fashion, FutureDet [25] directly predicts the future locations of objects observed at a specific time instead of predicting point-cloud sequences over time and then backcasting them to determine their origin in the current frame. This allows the model to reason about multiple possible futures by linking future and current locations in a many-to-one manner. This approach leverages existing LiDAR detectors to predict object positions in unseen future scans. Building upon the recently proposed CenterPoint LiDAR detector [17], FutureDet predicts not only future locations but also velocity vectors for each object in every frame between the current and final predicted future frame. This enables the model to estimate consistent object trajectories throughout the entire forecasting horizon. In the process of forecasting, it is essential to link all trajectories to the collection of object detections in the current (observed) LiDAR scan. For each future detection i, FutureDet computes the distance to every detection j from the previous timestep. Subsequently, for each i, FutureDet selects the most suitable j (permitting multiple-to-one matching).

Additionally, it is argued that current evaluation metrics for predicting directly from raw LiDAR data are inadequate as they can be manipulated by simplistic predictors, leading to inflated performance. These metrics, originally designed for trajectory-based prediction, do not effectively address the interconnected tasks of detection and forecasting. To overcome these limitations, a novel evaluation procedure is proposed by FutureDet. The new metric integrates both detection and forecasting tasks. Notably, this approach surpasses state-of-the-art methods without the necessity of object tracks or high definition (HD) maps as model inputs.

### 2.2. Neural Architecture Search

Optimizing model hyperparameters is an effective way to improve intelligent systems using automated machine learning (AutoML) [26]. Neural architecture search (NAS) is a subset of AutoML that aims to create efficient neural networks for complex learning tasks [27]. Early NAS methods used reinforcement learning (RL) [28,29] or evolutionary algorithms [30,31]. However, evaluating 20,000 neural architectures over four days requires remarkable computing capacity, such as 500 NVIDIA^®^ GPUs used in this study were sourced from NVIDIA Corporation, which is headquartered in Santa Clara, CA, USA [28]. Recently, methods for differentiable neural architecture search (NAS) have been proven to achieve state-of-the-art results across various learning tasks [32,33,34]. DARTS [33] is a differentiable NAS method that uses the gradient descent algorithm to search and train neural architecture cells jointly. Despite the success of differentiable NAS methods in various domains [34], they suffer from inefficient training due to interfering with the training of different sub-networks each other [35]. Moreover, it has been proven that with equal search spaces and training setups, differentiable NAS methods converge to similar results [36].

Meta-heuristic-based NAS methods [37,38,39] benefit from fast and flexible algorithms to search a discrete search space. FastStereoNet [39] is a state-of-the-art meta-heuristic method that designs an accurate depth estimation pipeline. TrajectoryNAS is a fast multi-objective meta-heuristic NAS designed to optimize trajectory prediction approaches by searching a wider design space compared to differentiable methods or evolutionary NAS approaches.

## 3. TrajectoryNAS

Current trajectory prediction techniques rely on handcrafted neural network architectures. These models, while effective for tasks like 3D object detection, are suboptimal for trajectory prediction. Building on the success of neural architecture search (NAS), TrajectoryNAS offers an interactive approach to designing neural networks specifically for 3D trajectory prediction. However, it is important to note that training a trajectory prediction model is both costly and time-consuming, requiring approximately 12 GPU hours for a single model.

As a result, the NAS procedure becomes significantly slow, requiring approximately 1200 GPU hours. To expedite the training process, we leverage state-of-the-art techniques (e.g.,  [40,41,42]) that utilize a miniaturized NuScenes dataset to reduce the computational demand for communication rescores. As an example, Blanch et al.  [42] demonstrates the use of a mini-dataset for hyperparameter optimization. Similarly, each model generated by neural architecture search (NAS) is trained on a standard mini-subset of the NuScenes dataset [5]. This technique reduces the evaluation time for each model to nearly 1 h, making the process approximately 12 times faster.

**Figure 2:**
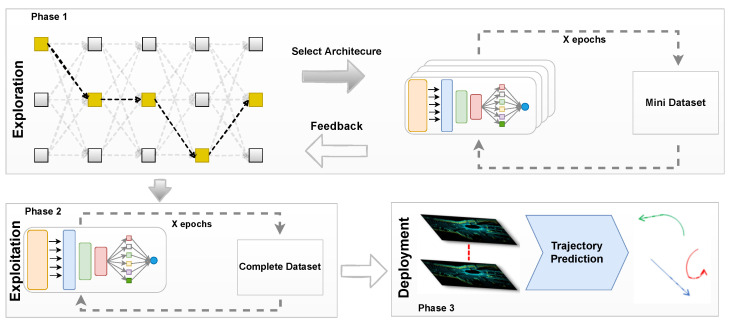
TrajectoryNAS state diagram. A model generated from the search space. The generated model trains using the mini dataset. The results are sent back to search space to generate a new model. The best final model is fully trained using the original dataset.

Figure 2 elaborates the TrajectoryNAS state diagram. The TrajectoryNAS workflow consists of three phases: Phase 1, exploration, where the metaheuristic algorithm suggests new architectures, and each architecture is trained using a mini dataset to compare with other suggested architectures. In Phase 2, the architecture with the highest accuracy on the mini dataset is retrained using the full-size dataset. Finally, in Phase 3, the fully trained model is deployed on hardware and tested with the test dataset to report the final results.

TrajectoryNAS is a one-stage trajectory prediction [21,25]. The model takes a sequence of Lidar data captured from the scene, which integrates a robust 3D backbone with cutting-edge neural architecture search (NAS) to refine map-view feature extraction from LiDAR point clouds. This innovative architecture further evolves by automating the design of multi-2D CNN detection heads, specifically tailored for future object detection and trajectory prediction. By detecting objects across multiple future timesteps and accurately projecting their movements back to the current moment, TrajectoryNAS stands out for its precision in trajectory prediction. This system not only anticipates the dynamic positioning of objects but also adjusts its computational strategies in real-time, ensuring a high degree of accuracy and efficiency in processing. The inclusion of NAS allows for continuous improvement of the detection and prediction heads, making TrajectoryNAS a highly adaptive and forward-thinking solution in the realm of autonomous navigation and surveillance technologies. TrajectoryNAS employs a hybrid optimization strategy to minimize optimization costs. The process is divided into two phases. The first phase, called exploration, involves the algorithm exploring various neural architecture designs to identify the optimal design. This phase is time-consuming as the algorithm must evaluate a wide range of parameters within the search space elaborated in Section 3.1. During the exploration phase, it is crucial to establish a comparative accuracy metric that can evaluate different architectures and determine the relative optimal design. To reduce processing time, the exploration phase utilizes a subset of the Nuscenes dataset [5], which contains significantly less data but maintains a distribution similar to the full dataset. To ensure that the selected model performs efficiently on the complete dataset, we use the full dataset in the second phase, known as exploitation, where we report the final accuracy of the designed model.

### 3.1. Search Space

**Figure 3:**
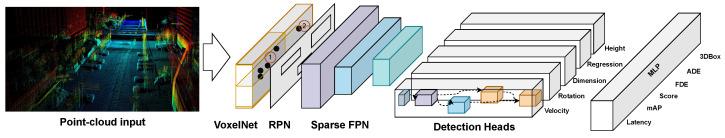
The overview of TrajcetoryNAS process.

TrajectoryNAS search space is demonstrated in Figure 3.

The TrajectryNAS architecture stands out as a solution for object detection and trajectory prediction, particularly in scenarios like autonomous driving, where understanding dynamic environments is paramount. It skillfully merges spatial and temporal object analyses, predicting not only the present state but also future trajectories.

#### 3.1.1. 3D Object Detection with VoxelNet

Modern 3D object detection methods [17,43,44] utilize a 3D encoder that converts the point cloud into regular bins. A point-based network [45] then extracts features from all the points within each bin. The 3D encoder subsequently pools these features to form its primary feature representation. Most of the computational workload is handled by the backbone network, which operates exclusively on these quantized and pooled feature representations. The output of the backbone network is a map-view feature map $M\in R^{W\times L\times F}$ with width *W*, length *L*, and *F* channels in a map-view reference frame. The width and height are directly related to the resolution of the individual voxel bins and the stride of the backbone network. Common backbone architectures include VoxelNet [46,47] and PointPillars [43]. This work employs VoxelNet as the backbone network.

VoxelNet is a novel approach for 3D object detection from LiDAR data and comprises three functional blocks:

Feature Learning Network: This network processes raw LiDAR data by dividing the point cloud into 3D voxels. A crucial component is the voxel feature encoding (VFE) layer, which transforms each group of points within a voxel into a unified feature representation. By stacking multiple VFE layers, the network learns complex features that capture local 3D shape information within the point cloud.

Convolutional Middle Layers: After the feature learning network generates a volumetric representation with encoded features, these features are further processed by 3D convolutional layers. These layers aggregate local voxel features, transforming the point-cloud data into a richer and more informative high-dimensional representation.

Region Proposal Network (RPN): The final stage utilizes an RPN [48] to generate 3D object detections. The input to the RPN is the feature map provided by the convolutional middle layers. The network consists of three blocks of fully convolutional layers, with batch normalization (BN) and ReLU operations applied after each layer. The output of each block is up-sampled to a fixed size and concatenated to construct a high-resolution feature map.

#### 3.1.2. Trajectory Prediction

TrajectoryNAS detects objects in both the current and future frames, projecting future detections back to the reference frame. We hypothesize that detecting objects in future frames requires the network to learn forecasted feature representations, as suggested by Peri et al. [25]. The network uses features extracted from the feature extraction module (VoxelNet) to predict features for the next timestep (t+1). After each prediction, a detection module refines the results. Initially, the extracted features are used for object detection in the current frame. Simultaneously, a copy of these features is passed to the prediction network to forecast features for the next timestep. This process is repeated iteratively, with predicted features being used for subsequent detection modules, until both the features and object detections are obtained for the final timestep.

Each detection module contains five parallel prediction heads, each responsible for a specific aspect of the object’s state: velocity, rotation, dimension, regression (bounding box refinement), and height. These heads work in concert to provide a comprehensive description of an object’s current position and orientation at time *t*.

To link objects across different frames, our network detects objects in both the current and future frames and predicts offsets to associate them back in time, assuming constant velocity between frames. Trajectory construction involves aligning all trajectories with the objects detected in the current LiDAR scan. Each detected object in the future frame (*i*) is matched to the previous frame (*j*) using the constant velocity equation. The distance between the detected object at time j and all other detected objects is calculated, and the closest object is then selected.

Such an approach allows TrajectryNAS to not only navigate but also anticipate complex dynamic behaviors, making it an invaluable asset in fields where predicting future states is crucial for proactive decision-making. This architecture’s ability to foresee the direction and movement of objects enriches scene understanding and enhances planning for autonomous systems, offering a comprehensive and forward-looking perspective on environmental dynamics.

The TrajectoryNAS system automatically designs the region proposal network (RPN) and the prediction heads using the aforementioned layers. It explores an expansive space of $2^{300}$ potential architectures to identify an optimal balance between speed and accuracy. This approach enables the selection of a highly efficient and accurate architecture tailored for specific applications.

### 3.2. Search Algorithm

To improve the accuracy of trajectory prediction while reducing network inference time, we employ the multi-objective simulated annealing (MOSA) algorithm, as described in [49]. The search algorithm optimizes the trajectory prediction in the design time and before training the model. The reason for using MOSA is its simplicity and its superior ability to explore a wide range of candidates compared to gradient-based algorithms. MOSA is also capable of finding global optima due to its effective exploration-exploitation balance. These attributes make MOSA a robust choice for optimizing complex, multi-objective problems such as trajectory prediction. MOSA selects candidates based on the probability of min(1,exp(−Δ/T)), where Δ is the energy difference between present and newly generated candidates, and *T* is the regulating parameter for annealing temperature. Initially, *T* starts from a large value ($T_{Max}$) and gradually decreases to a small value ($T_{Min}$). Setting $T_{Max}$ to a large value allows for exploration of non-optimal choices, while $T_{Min}$ being small gives the maximum selection chance to optimal candidates (exploitation).

To achieve this optimization, we use a multi-objective energy function (Equation (1)).

The energy function (*E*) is the product of the network *latency* (*t*) and the weighted mean average precision of the predicted future place of the object and its actual place (*mAP*), weighted average displacement (*ADE*) error, and weighted final displacement error (*FDE*).

(1) $$\begin{array}{c} E=Latency\times {mAP}^{\alpha }\times {ADE}^{\beta }\times {FDE}^{\gamma } \end{array}$$

where α, β, and γ are weights of *mAP*, *ADE*, and *FDE*, respectively. We do not use any proxy, such as Floating-Point-Operations-per-Second (FLOPs), for inference time estimation. Instead, we run the network directly on the target hardware (NVIDIA^®^ RTX A4000 were sourced from NVIDIA Corporation, which is headquartered in Santa Clara, CA, USA) to measure the exact inference time. Algorithm 1 is a complete description of the TrajectoryNAS flow.
**Algorithm 1** TrajecoryNAS1:**procedure** Exploration2:      M← *Mini-Dataset*3:      $A_{\mathrm{init}}\leftarrow$ *InitialArchitecture*4:      $T_{Max}\leftarrow$ MaximumTemperature5:      $T_{Min}\leftarrow$ MinimumTemperature6:      $T_{Factor}\leftarrow -Log\left(T_{Max},T_{Min}\right)$7:      $A_{\mathrm{best}}\leftarrow A_{\mathrm{Init}}$8:      $A_{\mathrm{current}}\leftarrow A_{\mathrm{Init}}$9:      train$\left(A_{\mathrm{current}},M\right)$10:     **for** each iteration *i* from 1 to MaxIterations **do**11:           $A_{\mathrm{new}}\leftarrow$ GenerateNeighbor($A_{\mathrm{current}}$)12:           $train(A_{\mathrm{new}},M)$13:           ΔE← E($A_{\mathrm{new}}$) − E($A_{\mathrm{current}}$)14:           **if** ΔE<0 **then**15:                 $A_{\mathrm{current}}\leftarrow A_{\mathrm{new}}$16:           **else**17:                 r← Random number in [0,1]18:                 **if** r<min(1,exp(−Δ/T)) **then**19:                       $A_{\mathrm{current}}\leftarrow A_{\mathrm{new}}$20:                 **end if**21:           **end if**22:           **if** E($A_{\mathrm{current}}$) < E($A_{\mathrm{best}}$) **then**23:                 $A_{\mathrm{best}}\leftarrow A_{\mathrm{current}}$24:           **end if**25:           $T\leftarrow T_{Max}\times Exp\left(T_{Factor}\times \left(i/MaxIterations\right)\right)$26:     **end for**27:     **return** $A_{\mathrm{best}}$28:**end procedure**29:**procedure** Exploitation($A_{\mathrm{best}}$)30:     C← *Complete-Dataset*31:     train$\left(A_{\mathrm{best}},C\right)$32:     accuracy← vaidate$\left(A_{\mathrm{best}},C\right)$33:     **return** accuracy34:**end procedure**

## 4. Experimental Setup

We demonstrate the effectiveness of our approach on a large-scale real-world driving dataset. We focus on modular metrics for detection and prediction, as well as system metrics for end-to-end perception and prediction.

### 4.1. Dataset

Our experimental analysis was performed on the nuScenes [5] dataset, which contains 1000 log snippets, each lasting 20 s. We utilized two officially released divisions of the dataset: the Mini and Trainval splits. The Mini split, which consists of 10 scenes, is a subset of the Trainval split. The Trainval split contains 700 scenes for training purposes and 150 scenes for validation. Additionally, the test split, containing 150 scenes, is designated for challenges and lacks object annotations.

### 4.2. Evaluation Metrics

We follow the detection and prediction metrics defined by [25] to have a fair comparison with other state-of-the-art. Specifically, we use average precision (${AP}_{det}$) for detection and future average precision (${AP}_{f}$) for trajectory prediction.

**Detection Average Precision (${AP}_{det}$):** ${AP}_{det}$ is defined as the area under the precision-recall curve [50], commonly averaged over multiple spatial overlap thresholds [51]. To compute AP, we first determine the set of true positives (TP) and false positives (FP) to evaluate precision and recall.

**Future Average Precision (${AP}_{f}$):** future Average Precision (${AP}_{f}$) is a metric used to evaluate the accuracy of future trajectory predictions anchored to detected objects in the current frame ($t_{obs}$). It penalizes incorrect future predictions (false predictions) and missed detection (missed predictions). A true positive (TP) requires a positive match both at the current timestamp ($t_{obs}$) and the final timestep ($t_{obs}$ + T), Otherwise, a prediction is considered to be a false positive (FP). A successful match in the current frame is determined based on distance thresholds of 0.5, 1, 2, 4 m for the current frame and 1, 2, 4, 8 m for the final timestep [25]. ${AP}_{f}$ considers all detections and penalizes missed predictions, typically measured by the miss rate.

We have defined three subclasses: static cars, linearly moving cars, and non-linearly moving cars [25], and we report ${AP}_{f}$ and ${AP}_{det}$ for these three classes. Subsequently, we evaluate the mean average precision for the future (${mAP}_{f}$) as follows: ${mAP}_{f}=1/3\times \left({AP}_{f}^{lin.}+{AP}_{f}^{non-lin.}+{AP}_{f}^{stat.}\right)$. Similarly, ${mAP}_{det}$ is evaluated as the average ${AP}_{det}$ over the three subclasses. Subclass labels are determined based on the trajectory (whether ground truth or predicted). First, we calculate the intersection over union (IoU) between the bounding boxes at the first and last timestep. If the IoU is greater than 0, the trajectory is labeled as static. Next, we use the velocity from the first timestep to project a target box. If the IoU between this target box and the last timestep box is greater than 0, the trajectory is labeled as linear. Trajectories that do not fit either category are labeled as non-linear.

${mAP}_{f}$ and ${mAP}_{det}$ provide a more realistic evaluation by jointly assessing detection and prediction accuracy. They penalize both missed predictions and false predictions, ensuring that only predictions correctly matched to detected objects are considered true positives [25]. This joint evaluation embraces the inherent multi-future nature of prediction and is robust against imbalanced data scenarios, such as the high proportion of stationary cars in the nuScenes dataset.

### 4.3. Configuration Setup

For this study, Table 1 provides a brief overview of the configuration setup.

## 5. Results

### 5.1. Trajectory Prediction Performance

As presented in Table 2 and Table 3, the comparison of car and pedestrian trajectory prediction results demonstrates that TrajectoryNAS outperforms other state-of-the-art trajectory prediction methods in numerous parameters for car trajectory prediction and the majority of parameters for pedestrian trajectory prediction. Notably, the latency of TrajectoryNAS is comparable to that of Fast and Furious [21] and better than FutureDet [25], while TrajectoryNAS provides superior future average precision ($AP_{f}$) across all conditions for both linear and non-linear trajectories of cars and pedestrians. Future average precision ($AP_{f}$) is a novel trajectory prediction performance metric proposed by FutureDet [25] and proved to be more precise in demonstrating trajectory performance in comparison to previous metrics.

For cars, while Fast and Furious and FutureDet offer a marginal improvement in specific aspects when compared with TrajectoryNAS, TrajectoryNAS significantly surpasses the state-of-the-art in most parameters. This is evidenced by its top performance in average precision for static, linear, and non-linear trajectories, as well as its mean average precision (mAP), both for single (K=1) and multiple (K=5) predictions. Specifically, TrajectoryNAS achieves the highest detection accuracy and future average precision in almost all scenarios, highlighting its robustness and efficacy in car trajectory prediction.

Similarly, for pedestrian trajectory prediction, TrajectoryNAS demonstrates outstanding performance, particularly in accurately predicting linear and non-linear movements. It not only achieves the highest average precision scores across various scenarios but also maintains competitive latency, underscoring its effectiveness in real-time applications.

In conclusion, TrajectoryNAS advances the field of trajectory prediction by offering a highly accurate and efficient model. Its ability to provide better future average precision under different conditions for both cars and pedestrians, coupled with its comparable latency to leading models, positions TrajectoryNAS as a superior choice for trajectory prediction in dynamic environments.

### 5.2. Analysing Search Methods

**Figure 4:**
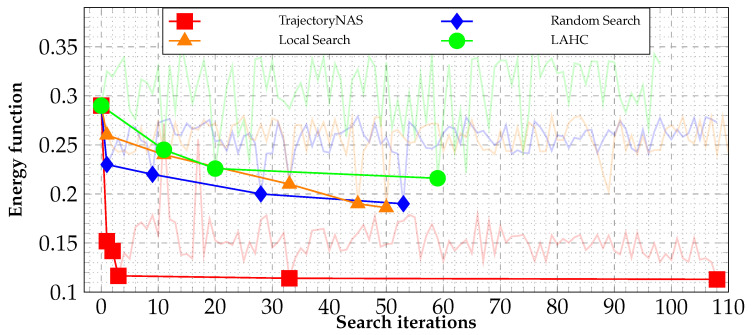
TrajectoryNAS optimization curve.

Figure 4 presents a detailed comparison of the energy function reduction (as defined in Equation (1)) during the search process employed by the TrajectoryNAS algorithm against those of random search and local search methods. This comparative analysis clearly demonstrates the limitations of both local search and random search techniques in effectively identifying the most optimal solution. Specifically, the best outcome identified through Random Search, characterized by an energy value of e = 0.19 as per Equation (1), was achieved in iteration 52. Similarly, Local Search’s most effective solution registered an energy value of e = 0.186, and this result was obtained in iteration 50.

Despite these efforts, both methods fall significantly short when compared to the capabilities of the TrajectoryNAS algorithm. TrajectoryNAS not only surpasses these traditional search methodologies in efficiently navigating towards more optimal solutions but also showcases its superiority by discovering an exceptionally lower energy value of 0.113. This landmark achievement was realized in iteration 108, underlining the algorithm’s advanced optimization prowess. Notably, the energy value associated with the best solution found by TrajectoryNAS is nearly half that of the best solutions unearthed by both random search and local search. This stark contrast underscores the advanced and sophisticated nature of TrajectoryNAS in exploring and exploiting the search space to find significantly more efficient solutions, thereby establishing a new benchmark in the quest for optimization within this context.

TrajectoryNAS overcomes Latent Acceptance Hill-Climbing (LAHC), a high-performance meta-heuristic algorithm as described by [52]. Figure 4 illustrates that LAHC becomes trapped in a local minimum and fails to escape to locate the global minimum. Consequently, the performance of LAHC is inferior to both local search and random search algorithms.

### 5.3. Visual Demonstration

**Figure 5:**
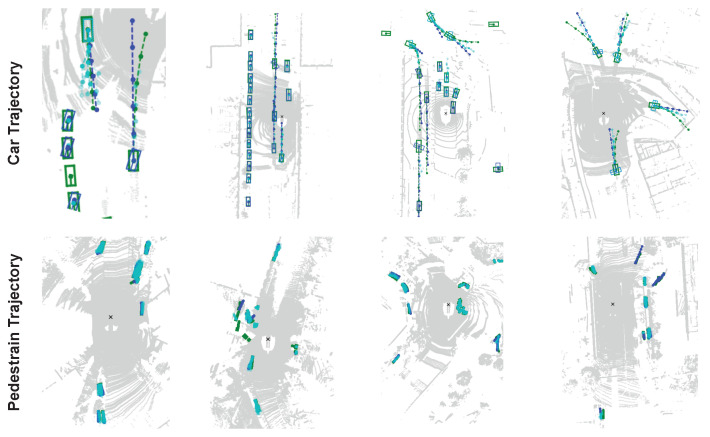
The visual demonstration of TrajectoryNAS; the first row is the trajectory prediction for cars, and the second row is the trajectory prediction for the pedestrian. Green lines are ground-truth. Blue lines are trajectory prediction with highest probability. Cyan lines are trajectory predictions with the highest probability.

The visual results of TrajectoryNAS are shown in Figure 5. As is evident, the results for both linear and non-linear activities for both cars and pedestrians closely match what occurs in the future. TrajectoryNAS is highly accurate in determining static and dynamic objects, and it rarely draws dynamic lines for static objects.

## 6. Conclusions

Trajectory prediction is one of the most important components of autonomous driving systems. A well-designed trajectory prediction model can accurately predict the trajectories of surrounding objects near the ego vehicle within an acceptable inference time, helping to prevent collisions by ensuring the ego vehicle avoids crossing their paths. State-of-the-art trajectory prediction models suffer from their handcrafted design, which leads to suboptimal accuracy and latency.

To resolve this problem, we propose TrajectoryNAS, a neural architecture search approach tailored for trajectory prediction applications, which designs accurate and low-latency trajectory prediction models using metaheuristic algorithms. Our empirical studies demonstrate that TrajectoryNAS achieves a minimum of 4.8% higher accuracy in predicting the trajectories of objects with non-linear paths. This highlights its effectiveness in predicting the trajectories of objects with more freedom of movement than vehicles, such as pedestrians. Our future work involves enhancing TrajectoryNAS to support novel deep learning approaches, such as vision transformers (ViTs), which have been well-demonstrated in meeting autonomous driving requirements, such as long-range perception.

## Figures and Tables

**Table 1:** Summarizing hardware specification, train, and search parameters.

Train/Test Hardware Device	Specification
GPU	NVIDIA^®^ RTX A4000
GPU Compiler	CUDA v11.7 & cuDNN v8.2.0
DL Framework	PyTorch v1.9.1
**Training and Search Parameters**	**Value**
Full-Training Epochs	20
Batch Size	1
Learning Rate	$5\times 10^{-4}$
Optimizer	Adam
$T_{Max}$/$T_{Min}$	2500/2.5

**Table 2:** Comparison TrajcetoryNAS and state-of-the-art trajectory prediction model on cars according to accuracy and latency metrics.

Method	Time (ms)	K=1								K=5							
		${\mathrm{AP}}^{\mathrm{stat}.}$		${\mathrm{AP}}^{\mathrm{lin}.}$		${\mathrm{AP}}^{\mathrm{non}-\mathrm{lin}.}$		mAP		${\mathrm{AP}}^{\mathrm{stat}.}$		${\mathrm{AP}}^{\mathrm{lin}.}$		${\mathrm{AP}}^{\mathrm{non}-\mathrm{lin}.}$		mAP	
		${\mathrm{AP}}_{\det.}$	${\mathrm{AP}}_{f}$	${\mathrm{AP}}_{\det.}$	${\mathrm{AP}}_{f}$	${\mathrm{AP}}_{\det.}$	${\mathrm{AP}}_{f}$	${\mathrm{AP}}_{\det.}$	${\mathrm{AP}}_{f}$	${\mathrm{AP}}_{\det.}$	${\mathrm{AP}}_{f}$	${\mathrm{AP}}_{\det.}$	${\mathrm{AP}}_{f}$	${\mathrm{AP}}_{\det.}$	${\mathrm{AP}}_{f}$	${\mathrm{AP}}_{\det.}$	${\mathrm{AP}}_{f}$
Detection + Constant Velocity	21	70.3	66.0	65.8	21.2	90.0	6.5	75.4	31.12	70.3	66.0	65.8	21.2	90.0	6.5	75.4	31.2
Detection + Forecast [21]	20	69.1	64.7	66.1	22.2	86.3	7.5	73.8	31.5	69.1	64.7	66.1	22.2	86.3	7.5	73.8	31.5
FutureDet [25]	24	70.0	65.5	62.9	24.9	91.8	10.1	74.9	33.5	70.1	67.3	62.9	27.7	91.7	11.7	74.9	35.6
TrajectoryNAS (ours)	**22**	**71.0**	**65.6**	**63.8**	**26**	**91.2**	**10.3**	**75**	**34**	**71**	**67.4**	**63.8**	**29.2**	**91.1**	**12.1**	**75.3**	**36.2**

**Table 3:** Comparison TrajcetoryNAS and state-of-the-art trajectory prediction model on pedestrian according to accuracy and latency metrics.

Method	Time (ms)	K=1								K=5							
		${\mathrm{AP}}^{\mathrm{stat}.}$		${\mathrm{AP}}^{\mathrm{lin}.}$		${\mathrm{AP}}^{\mathrm{non}-\mathrm{lin}.}$		mAP		${\mathrm{AP}}^{\mathrm{stat}.}$		${\mathrm{AP}}^{\mathrm{lin}.}$		${\mathrm{AP}}^{\mathrm{non}-\mathrm{lin}.}$		mAP	
		${\mathrm{AP}}_{\det.}$	${\mathrm{AP}}_{f}$	${\mathrm{AP}}_{\det.}$	${\mathrm{AP}}_{f}$	${\mathrm{AP}}_{\det.}$	${\mathrm{AP}}_{f}$	${\mathrm{AP}}_{\det.}$	${\mathrm{AP}}_{f}$	${\mathrm{AP}}_{\det.}$	${\mathrm{AP}}_{f}$	${\mathrm{AP}}_{\det.}$	${\mathrm{AP}}_{f}$	${\mathrm{AP}}_{\det.}$	${\mathrm{AP}}_{f}$	${\mathrm{AP}}_{\det.}$	${\mathrm{AP}}_{f}$
Detection + Constant Velocity	21	55.1	33.3	73.5	27.8	96.9	12.4	75.2	25.5	55.1	33.3	73.5	27.8	96.9	12.4	75.2	24.5
Detection + Forecast [21]	20	53.7	35.0	73.9	30.8	97.2	13.3	74.9	26.4	53.7	35.0	73.9	30.8	97.2	13.3	74.9	26.4
FutureDet [25]	24	53.1	33.3	72.4	32.6	95.2	14.7	73.6	26.9	53.1	35.1	72.4	34.0	95.2	15.0	73.6	28.0
TrajectoryNAS (ours)	**22**	**55.8**	**37.1**	**77.9**	**39.9**	**95.2**	**17.7**	**76.3**	**31.3**	**55.8**	**38.6**	**77.9**	**40.9**	**95.2**	**17.9**	**76.3**	**32.5**

## Data Availability

Data available on request due to restrictions (e.g., privacy, legal or ethical reasons).

## References

[1] Liang M., Yang B., Zeng W., Chen Y., Hu R., Casas S., Urtasun R. Pnpnet: End-to-end perception and prediction with tracking in the loop. Proceedings of the IEEE/CVF Conference on Computer Vision and Pattern Recognition.

[2] Li L.L., Yang B., Liang M., Zeng W., Ren M., Segal S., Urtasun R. End-to-end contextual perception and prediction with interaction transformer. Proceedings of the 2020 IEEE/RSJ International Conference on Intelligent Robots and Systems (IROS).

[3] Marchetti F., Becattini F., Seidenari L., Del Bimbo A. (2020). Multiple trajectory prediction of moving agents with memory augmented networks. IEEE Trans. Pattern Anal. Mach. Intell..

[4] Charroud A., El Moutaouakil K., Palade V., Yahyaouy A., Onyekpe U., Eyo E.U. (2024). Localization and Mapping for Self-Driving Vehicles: A Survey. Machines.

[5] Caesar H., Bankiti V., Lang A.H., Vora S., Liong V.E., Xu Q., Krishnan A., Pan Y., Baldan G., Beijbom O. (2019). nuScenes: A multimodal dataset for autonomous driving. arXiv.

[6] Chang M.F., Lambert J.W., Sangkloy P., Singh J., Bak S., Hartnett A., Wang D., Carr P., Lucey S., Ramanan D. Argoverse: 3D Tracking and Forecasting with Rich Maps. Proceedings of the Conference on Computer Vision and Pattern Recognition (CVPR).

[7] Leon F., Gavrilescu M. (2021). A review of tracking and trajectory prediction methods for autonomous driving. Mathematics.

[8] Phan-Minh T., Grigore E.C., Boulton F.A., Beijbom O., Wolff E.M. Covernet: Multimodal behavior prediction using trajectory sets. Proceedings of the IEEE/CVF Conference on Computer Vision and Pattern Recognition.

[9] Gao J., Sun C., Zhao H., Shen Y., Anguelov D., Li C., Schmid C. Vectornet: Encoding hd maps and agent dynamics from vectorized representation. Proceedings of the IEEE/CVF Conference on Computer Vision and Pattern Recognition.

[10] Liang M., Yang B., Hu R., Chen Y., Liao R., Feng S., Urtasun R. (2020). Learning lane graph representations for motion forecasting. Proceedings of the Computer Vision–ECCV 2020: 16th European Conference, Glasgow, UK, 23–28 August 2020.

[11] Ye M., Cao T., Chen Q. Tpcn: Temporal point cloud networks for motion forecasting. Proceedings of the IEEE/CVF Conference on Computer Vision and Pattern Recognition.

[12] Han K., Wang Y., Chen H., Chen X., Guo J., Liu Z., Tang Y., Xiao A., Xu C., Xu Y. (2022). A survey on vision transformer. IEEE Trans. Pattern Anal. Mach. Intell..

[13] Yuan Y., Weng X., Ou Y., Kitani K.M. Agentformer: Agent-aware transformers for socio-temporal multi-agent forecasting. Proceedings of the IEEE/CVF International Conference on Computer Vision.

[14] Khandelwal S., Qi W., Singh J., Hartnett A., Ramanan D. (2020). What-if motion prediction for autonomous driving. arXiv.

[15] Weng X., Ivanovic B., Kitani K., Pavone M. Whose track is it anyway? Improving robustness to tracking errors with affinity-based trajectory prediction. Proceedings of the IEEE/CVF Conference on Computer Vision and Pattern Recognition.

[16] Wang S., Sun Y., Liu C., Liu M. (2020). Pointtracknet: An end-to-end network for 3-d object detection and tracking from point clouds. IEEE Robot. Autom. Lett..

[17] Yin T., Zhou X., Krahenbuhl P. Center-based 3d object detection and tracking. Proceedings of the IEEE/CVF Conference on Computer Vision and Pattern Recognition.

[18] Li X., Guivant J.E. (2023). Efficient and Accurate Object Detection With Simultaneous Classification and Tracking Under Limited Computing Power. IEEE Trans. Intell. Transp. Syst..

[19] Simon M., Amende K., Kraus A., Honer J., Samann T., Kaulbersch H., Milz S., Michael Gross H. Complexer-yolo: Real-time 3d object detection and tracking on semantic point clouds. Proceedings of the IEEE/CVF Conference on Computer Vision and Pattern Recognition Workshops.

[20] Weng X., Yuan Y., Kitani K. (2021). PTP: Parallelized tracking and prediction with graph neural networks and diversity sampling. IEEE Robot. Autom. Lett..

[21] Luo W., Yang B., Urtasun R. Fast and furious: Real time end-to-end 3d detection, tracking and motion forecasting with a single convolutional net. Proceedings of the IEEE Conference on Computer Vision and Pattern Recognition.

[22] Casas S., Luo W., Urtasun R. Intentnet: Learning to predict intention from raw sensor data. Proceedings of the Conference on Robot Learning PMLR.

[23] Zeng W., Luo W., Suo S., Sadat A., Yang B., Casas S., Urtasun R. End-to-end interpretable neural motion planner. Proceedings of the IEEE/CVF Conference on Computer Vision and Pattern Recognition.

[24] Weng X., Wang J., Levine S., Kitani K., Rhinehart N. Inverting the pose forecasting pipeline with SPF2: Sequential pointcloud forecasting for sequential pose forecasting. Proceedings of the Conference on Robot Learning PMLR.

[25] Peri N., Luiten J., Li M., Ošep A., Leal-Taixé L., Ramanan D. Forecasting from lidar via future object detection. Proceedings of the IEEE/CVF Conference on Computer Vision and Pattern Recognition.

[26] He X., Zhao K., Chu X. (2021). AutoML: A Survey of the State-of-the-Art. Knowl.-Based Syst..

[27] Elsken T., Metzen J.H., Hutter F. (2019). Neural architecture search: A survey. J. Mach. Learn. Res..

[28] Zoph B., Le Q.V. (2016). Neural architecture search with reinforcement learning. arXiv.

[29] Hsu C.H., Chang S.H., Liang J.H., Chou H.P., Liu C.H., Chang S.C., Pan J.Y., Chen Y.T., Wei W., Juan D.C. (2018). Monas: Multi-objective neural architecture search using reinforcement learning. arXiv.

[30] Loni M., Sinaei S., Zoljodi A., Daneshtalab M., Sjödin M. (2020). DeepMaker: A multi-objective optimization framework for deep neural networks in embedded systems. Microprocess. Microsyst..

[31] Loni M., Zoljodi A., Sinaei S., Daneshtalab M., Sjödin M. Neuropower: Designing energy efficient convolutional neural network architecture for embedded systems. Proceedings of the International Conference on Artificial Neural Networks.

[32] Liu C., Chen L.C., Schroff F., Adam H., Hua W., Yuille A.L., Fei-Fei L. Auto-deeplab: Hierarchical neural architecture search for semantic image segmentation. Proceedings of the IEEE/CVF Conference on Computer Vision and Pattern Recognition.

[33] Liu H., Simonyan K., Yang Y. (2018). Darts: Differentiable architecture search. arXiv.

[34] Loni M., Mousavi H., Riazati M., Daneshtalab M., Sjödin M. TAS:Ternarized Neural Architecture Search for Resource-Constrained Edge Devices. Proceedings of the Design, Automation & Test in Europe Conference & Exhibition DATE’22.

[35] Cai H., Gan C., Wang T., Zhang Z., Han S. (2019). Once-for-all: Train one network and specialize it for efficient deployment. arXiv.

[36] Dong X., Liu L., Musial K., Gabrys B. (2021). NATS-Bench: Benchmarking NAS Algorithms for Architecture Topology and Size. IEEE Trans. Pattern Anal. Mach. Intell..

[37] Loni M., Zoljodi A., Maier D., Majd A., Daneshtalab M., Sjödin M., Juurlink B., Akbari R. DenseDisp: Resource-Aware Disparity Map Estimation by Compressing Siamese Neural Architecture. Proceedings of the 2020 IEEE Congress on Evolutionary Computation (CEC).

[38] Xu H., Wang S., Cai X., Zhang W., Liang X., Li Z. (2020). Curvelane-nas: Unifying lane-sensitive architecture search and adaptive point blending. Proceedings of the Computer Vision—ECCV 2020: 16th European Conference, Glasgow, UK, 23–28 August 2020.

[39] Loni M., Zoljodi A., Majd A., Ahn B.H., Daneshtalab M., Sjödin M., Esmaeilzadeh H. (2021). FastStereoNet: A Fast Neural Architecture Search for Improving the Inference of Disparity Estimation on Resource-Limited Platforms. IEEE Trans. Syst. Man Cybern. Syst..

[40] Xie S., Li Z., Wang Z., Xie C. (2023). On the adversarial robustness of camera-based 3d object detection. arXiv.

[41] Kälble J., Wirges S., Tatarchenko M., Ilg E. Accurate Training Data for Occupancy Map Prediction in Automated Driving Using Evidence Theory. Proceedings of the IEEE/CVF Conference on Computer Vision and Pattern Recognition.

[42] Blanch M.R., Li Z., Escalera S., Nasrollahi K. LiDAR-Assisted 3D Human Detection for Video Surveillance. Proceedings of the IEEE/CVF Winter Conference on Applications of Computer Vision.

[43] Lang A.H., Vora S., Caesar H., Zhou L., Yang J., Beijbom O. Pointpillars: Fast encoders for object detection from point clouds. Proceedings of the IEEE/CVF Conference on Computer Vision and Pattern Recognition.

[44] He C., Zeng H., Huang J., Hua X.S., Zhang L. Structure aware single-stage 3d object detection from point cloud. Proceedings of the IEEE/CVF Conference on Computer Vision and Pattern Recognition.

[45] Qi C.R., Su H., Mo K., Guibas L.J. Pointnet: Deep learning on point sets for 3d classification and segmentation. Proceedings of the IEEE Conference on Computer Vision and Pattern Recognition.

[46] Yan Y., Mao Y., Li B. (2018). Second: Sparsely embedded convolutional detection. Sensors.

[47] Zhou Y., Tuzel O. Voxelnet: End-to-end learning for point cloud based 3d object detection. Proceedings of the IEEE Conference on Computer Vision and Pattern Recognition.

[48] Ren S., He K., Girshick R., Sun J. (2016). Faster R-CNN: Towards real-time object detection with region proposal networks. IEEE Trans. Pattern Anal. Mach. Intell..

[49] Amine K. (2019). Multiobjective simulated annealing: Principles and algorithm variants. Adv. Oper. Res..

[50] Everingham M., Van Gool L., Williams C.K., Winn J., Zisserman A. (2010). The pascal visual object classes (voc) challenge. Int. J. Comput. Vis..

[51] Lin T.Y., Maire M., Belongie S., Hays J., Perona P., Ramanan D., Dollár P., Zitnick C.L. (2014). Microsoft coco: Common objects in context. Proceedings of the Computer Vision–ECCV 2014: 13th European Conference, Zurich, Switzerland, 6–12 September 2014.

[52] Burke E.K., Bykov Y. (2017). The late acceptance Hill-Climbing heuristic. Eur. J. Oper. Res..

